# Supplementary material for: microbeMASST: a taxonomically informed mass spectrometry search tool for microbial metabolomics data
Source: Nat Microbiol. 2024 Feb 5;9(2):336–45. doi: 10.1038/s41564-023-01575-9 (PMC10847041; doi:10.1038/s41564-023-01575-9)
Supplement: Supplementary file 1 — Supplementary Figs. 1–5. [file 41564_2023_1575_MOESM1_ESM.pdf]

nature microbiology

Brief Communication

https://doi.org/10.1038/s41564-023-01575-9

microbeMASST: a taxonomically informed mass spectrometry search tool for microbial metabolomics data

In the format provided by the authors and unedited

Supplementary Figures

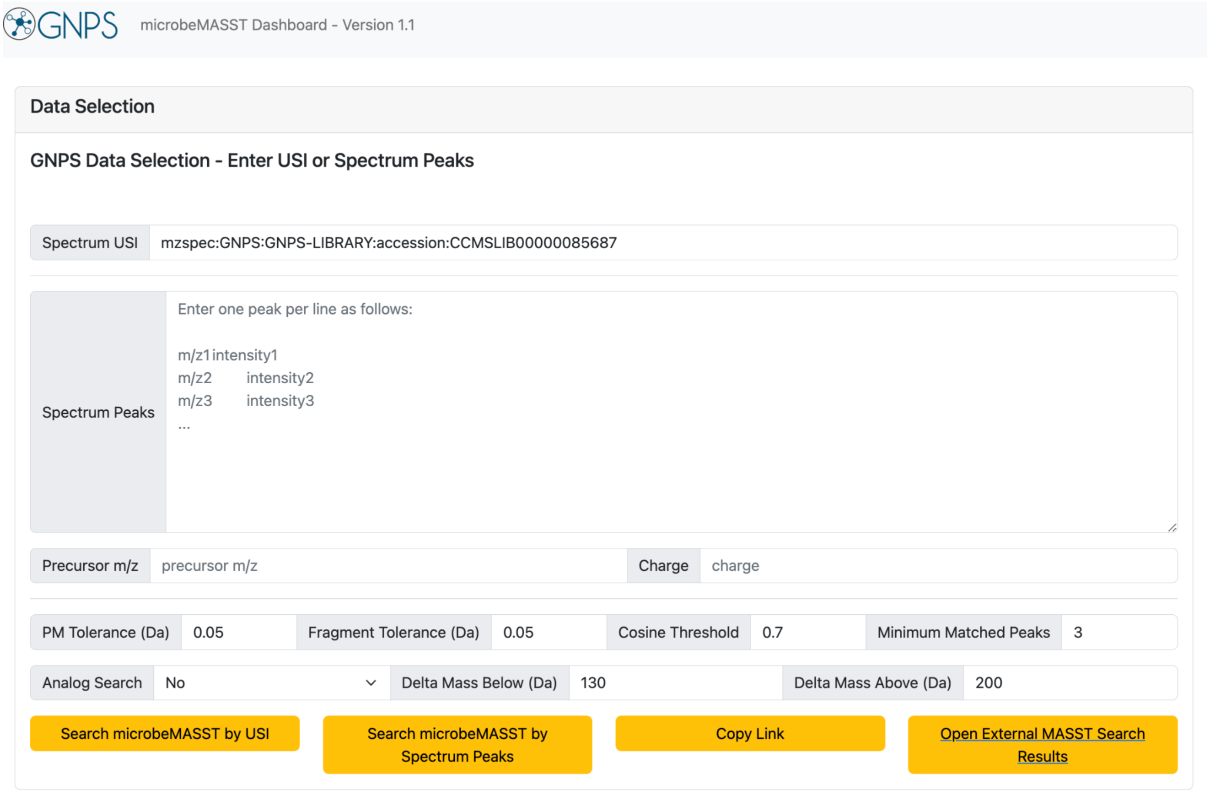

Supplementary Figure 1. microbeMASST web app

Users can access the microbeMASST web app at https://masst.gnps2.org/microbemasst/. They can search for MS/MS spectrum against the microbeMASST reference database by either providing a USI in `Spectrum USI` or by inputting fragment ions and their intensities and the precursor mass in `Spectrum Peak' and `Precursor m/z` respectively. Search parameters, such as parent ion and fragment ions tolerances, cosine threshold, and minimum matching peaks can be modified. Analog search can also be enabled. Finally, to submit a search query, users have just to click either on `Search microbeMASST by USI` or `Search microbeMASST by Spectrum Peak` based on the information that they have provided. Search jobs can be easily shared by clicking on `Copy Link`.

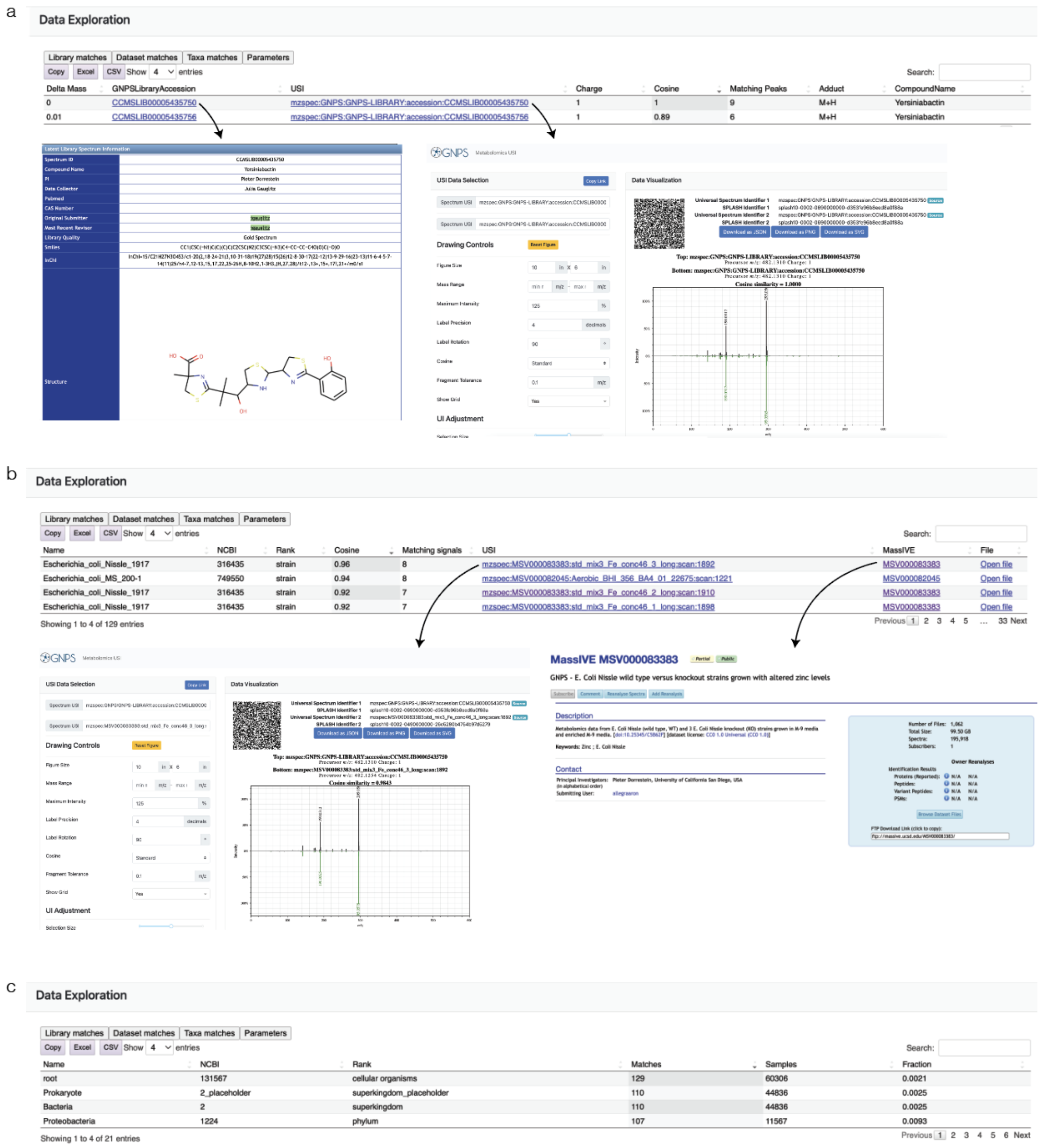

Supplementary Figure 2. Complementary output of microbeMASST

a) The queried MS/MS spectrum is searched against the GNPS libraries and, if matches are found, possible annotations are returned. Information on cosine similarity and number of matching peaks is provided and users can explore the associated GNPS Library Spectrum page and inspect mirror plots. **b)** Information of matching scans in the sample from the different taxa is provided. In addition to cosine similarity score, matching fragments, and the possibility to inspect mirror plots, users can retrieve the MassIVE ascension number of the project together with contact information on who deposited the data. **c)** Number of matches for each taxonomic level are returned. Users can observe how many samples for that specific taxon are part of microbeMASST and see how broadly the molecule is distributed within it (fraction).

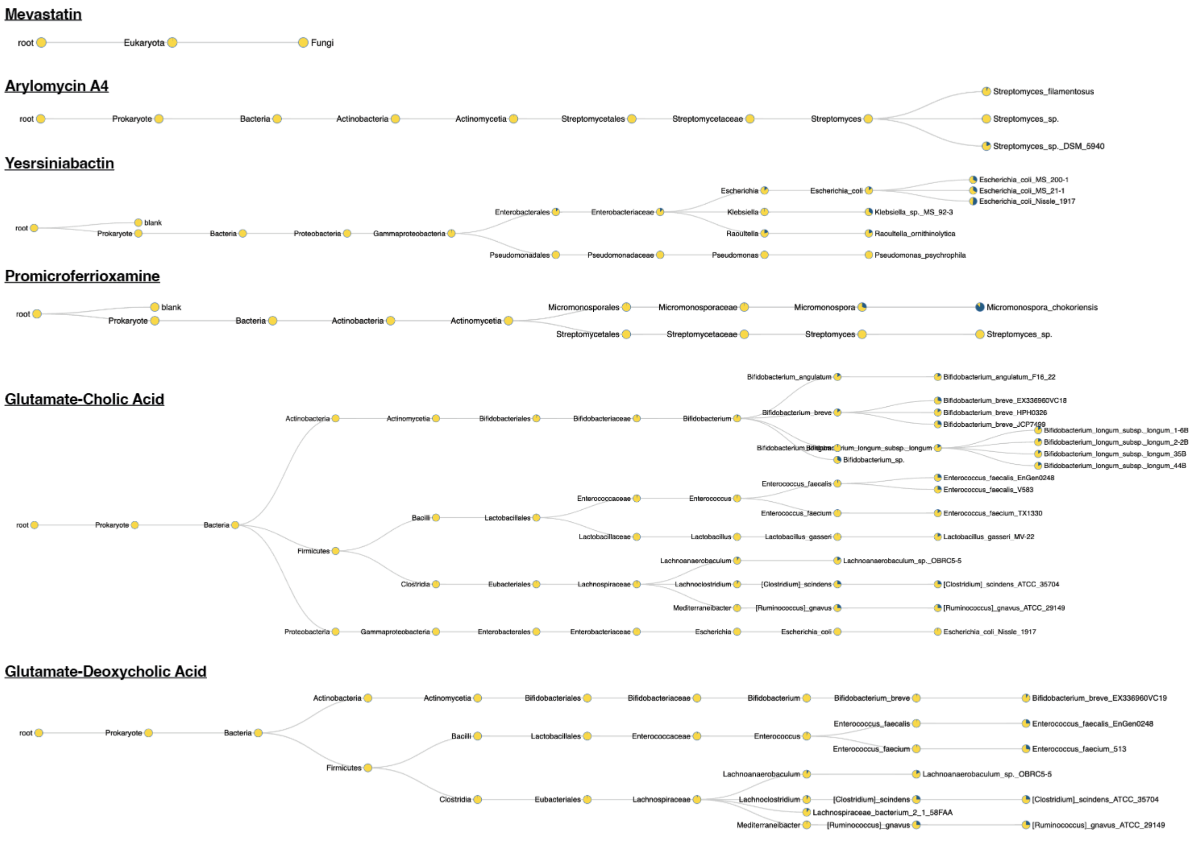

Supplementary Figure 3. Examples of microbeMASST outputs

Additional examples of results obtained from searching from mevastatin, arylomycin A4, yersiniabactin, promicroferrioxamine, glutamate-cholic acid, and glutamate deoxycholic acid. In all cases the molecules were found in the monocultures of known producers and not in human cell lines, confirming that microbeMASST can be used to search for microbial-derived molecules.

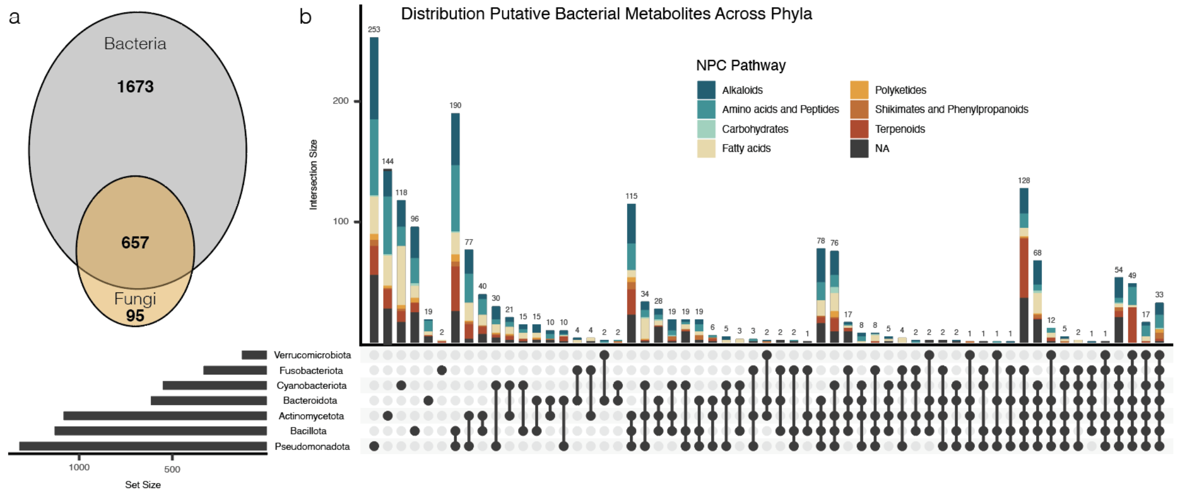

Supplementary Figure 4. Distribution putative microbial metabolites across phyla

a) Of the 2,425 MS/MS spectra that had a match exclusively to microbial monocultures, 1,673 were found only in bacteria, 95 in fungi, and 657 in both. b) Chemical classes were then predicted using SIRIUS and CANOPUS and their distribution across the different phyla was visualized using an Upset plot.

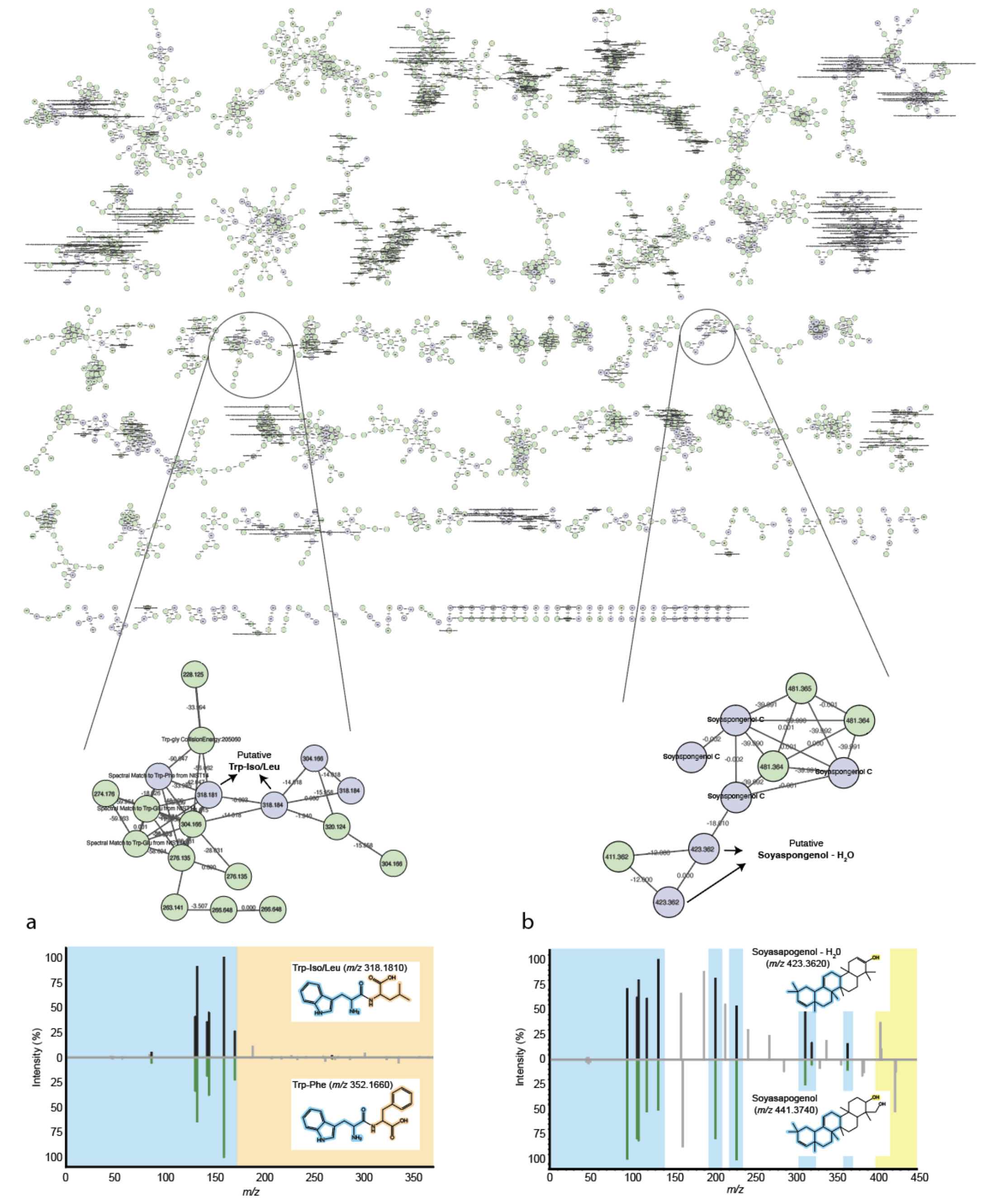

Supplementary Figure 5. Contextualization of the molecular network

The selected 512 MS/MS spectra were mapped back to the full molecular network generated from the data acquired from GF and SPF mice (MSV000079949). Only molecular families containing at least one of the MS/MS spectra of interest were retained and used to generate putative annotations of the unannotated spectra of interest. Each node represents a spectrum (ion), light blue indicates MS/MS of interest while green indicates spectra that were not retained in the performed downstream analysis. Within each node the *m/z* of the precursor ion is indicated, while on each edge the *m/z* difference between two connected nodes is reported. a) Ions with precursor mass 318.181 and directly connected to a spectrum annotated as the dipeptide Trp-Phe (*m/z* 352.144) can be tentatively annotated as Trp-Iso/Leu. b) Ions with precursor mass 423.362 connected to Soyasapogenol C, which can represent a soyasapogenol molecule with a loss of water (delta mass 18.01). Abbreviations: Trp, tryptophan; Phe, phenylalanine; Iso, isoleucine; Leu, leucine.
