# Supplementary material for: microbeMASST: a taxonomically informed mass spectrometry search tool for microbial metabolomics data
Source: Nat Microbiol. 2024 Feb 5;9(2):336–45. doi: 10.1038/s41564-023-01575-9 (PMC10847041; doi:10.1038/s41564-023-01575-9)
Supplement: Supplementary file 2 — Reporting Summary [file 41564_2023_1575_MOESM2_ESM.pdf]

nature portfolio

Corresponding author(s): Pieter Dorrestein

Last updated by author(s): Nov 27, 2023

Reporting Summary

Nature Portfolio wishes to improve the reproducibility of the work that we publish. This form provides structure for consistency and transparency in reporting. For further information on Nature Portfolio policies, see our Editorial Policies and the Editorial Policy Checklist.

Statistics

For all statistical analyses, confirm that the following items are present in the figure legend, table legend, main text, or Methods section.

n/a	Confirmed	
☒	☐	The exact sample size (*n*) for each experimental group/condition, given as a discrete number and unit of measurement
☒	☐	A statement on whether measurements were taken from distinct samples or whether the same sample was measured repeatedly
☒	☐	The statistical test(s) used AND whether they are one- or two-sided<br>*Only common tests should be described solely by name; describe more complex techniques in the Methods section.*
☒	☐	A description of all covariates tested
☒	☐	A description of any assumptions or corrections, such as tests of normality and adjustment for multiple comparisons
☒	☐	A full description of the statistical parameters including central tendency (e.g. means) or other basic estimates (e.g. regression coefficient) AND variation (e.g. standard deviation) or associated estimates of uncertainty (e.g. confidence intervals)
☒	☐	For null hypothesis testing, the test statistic (e.g. *F*, *t*, *r*) with confidence intervals, effect sizes, degrees of freedom and *P* value noted<br>*Give P values as exact values whenever suitable.*
☒	☐	For Bayesian analysis, information on the choice of priors and Markov chain Monte Carlo settings
☒	☐	For hierarchical and complex designs, identification of the appropriate level for tests and full reporting of outcomes
☒	☐	Estimates of effect sizes (e.g. Cohen's *d*, Pearson's *r*), indicating how they were calculated

Our web collection on statistics for biologists contains articles on many of the points above.

Software and code

Policy information about availability of computer code

Data collection	Data extracted from the open access GNPS/MassIVE database using ReDU (update Dec 2021) and my manually inspecting entries in the MassIVE website
Data analysis	MicrobeMASST code is available at https://github.com/robinschmid/microbe_masst. Code of microbeMASST web application is available at https://github.com/mwang87/GNPS_MASST/blob/master/dash_microbemasst.py. Code related to microbeMASST manuscript analysis is available at https://github.com/simonezuffa/Manuscript_microbeMASST. Molecular networking was performed through GNPS (https://gnps.ucsd.edu/ProteoSAFe/static/gnps-splash.jsp) and metadata was gathered using ReDU (https://redu.ucsd.edu/). the Fast Search Tool is available at https://fasst.gnps2.org/fastsearch/. Used NCBI tools can be found at https://www.ncbi.nlm.nih.gov/Taxonomy/TaxIdentifier/tax_identifier.cgi and https://www.ncbi.nlm.nih.gov/Taxonomy/Browser/wwwtax.cgi. MicrobeMASST code and data analysis code are based on R 4.2.2 (R Foundation for Statistical Computing) and Python 3.10 (Python Software Foundation).

For manuscripts utilizing custom algorithms or software that are central to the research but not yet described in published literature, software must be made available to editors and reviewers. We strongly encourage code deposition in a community repository (e.g. GitHub). See the Nature Portfolio guidelines for submitting code & software for further information.

Data

Policy information about availability of data

All manuscripts must include a data availability statement. This statement should provide the following information, where applicable:

- Accession codes, unique identifiers, or web links for publicly available datasets
- A description of any restrictions on data availability
- For clinical datasets or third party data, please ensure that the statement adheres to our policy

Data used to generate the reference database of microbeMASST are publicly available at GNPS/MassIVE (https://massive.ucsd.edu/). A list with the 537 accession codes (MassIVE IDs) of all the studies used to generate the reference database of this tool is available in Supplementary Table 2.

Research involving human participants, their data, or biological material

Policy information about studies with human participants or human data. See also policy information about sex, gender (identity/presentation), and sexual orientation and race, ethnicity and racism.

Reporting on sex and gender	No sex or gender are reported in the manuscript. A list of public datasets with data acquired from human biosamples that presented matches to the putative microbial MS/MS spectra of interest is available in Supplementary Table 3.
Reporting on race, ethnicity, or other socially relevant groupings	No race, ethnicity, or other socially relevant groupings are reported in the manuscript. A list of public datasets with data acquired from human biosamples that presented matches to the putative microbial MS/MS spectra of interest is available in Supplementary Table 3.
Population characteristics	No population characteristics is reported in the manuscript. A list of public datasets with data acquired from human biosamples that presented matches to the putative microbial MS/MS spectra of interest is available in Supplementary Table 3.
Recruitment	No recruitment is reported in the manuscript. A list of public datasets with data acquired from human biosamples that presented matches to the putative microbial MS/MS spectra of interest is available in Supplementary Table 3.
Ethics oversight	No ethics oversight is reported in the manuscript. A list of public datasets with data acquired from human biosamples that presented matches to the putative microbial MS/MS spectra of interest is available in Supplementary Table 3.

Note that full information on the approval of the study protocol must also be provided in the manuscript.

Field-specific reporting

Please select the one below that is the best fit for your research. If you are not sure, read the appropriate sections before making your selection.

☒ Life sciences ☐ Behavioural & social sciences ☐ Ecological, evolutionary & environmental sciences

For a reference copy of the document with all sections, see nature.com/documents/nr-reporting-summary-flat.pdf

Life sciences study design

All studies must disclose on these points even when the disclosure is negative.

Sample size	No sample size calculation was performed. All available data acquired from microbial monocultures deposited in GNPS/MassIVE were used to generate the microbeMASST reference database
Data exclusions	No data was excluded from the analysis
Replication	No biological replication required. Downstream data analysis can be replicated using publicly available code
Randomization	No randomization required and covariates were not controlled as not relevant to the study. Generate reference database encompass all relevant data deposited in GNPS/MassIVE and search tool is used to find matches between MS/MS spectra
Blinding	Blinding was not relevant to this study as we created a search tool.

Reporting for specific materials, systems and methods

We require information from authors about some types of materials, experimental systems and methods used in many studies. Here, indicate whether each material, system or method listed is relevant to your study. If you are not sure if a list item applies to your research, read the appropriate section before selecting a response.

Materials & experimental systems

n/a	Involved in the study
☒	☐ Antibodies
☒	☐ Eukaryotic cell lines
☒	☐ Palaeontology and archaeology
☒	☐ Animals and other organisms
☒	☐ Clinical data
☒	☐ Dual use research of concern
☒	☐ Plants

Methods

n/a	Involved in the study
☒	☐ ChIP-seq
☒	☐ Flow cytometry
☒	☐ MRI-based neuroimaging

Plants

Seed stocks	*Report on the source of all seed stocks or other plant material used. If applicable, state the seed stock centre and catalogue number. If plant specimens were collected from the field, describe the collection location, date and sampling procedures.*
Novel plant genotypes	*Describe the methods by which all novel plant genotypes were produced. This includes those generated by transgenic approaches, gene editing, chemical/radiation-based mutagenesis and hybridization. For transgenic lines, describe the transformation method, the number of independent lines analyzed and the generation upon which experiments were performed. For gene-edited lines, describe the editor used, the endogenous sequence targeted for editing, the targeting guide RNA sequence (if applicable) and how the editor was applied.*
Authentication	*Describe any authentication procedures for each seed stock used or novel genotype generated. Describe any experiments used to assess the effect of a mutation and, where applicable, how potential secondary effects (e.g. second site T-DNA insertions, mosiacism, off-target gene editing) were examined.*
